# Pseudomyxoma Peritonei: Presentation of Two Cases and Challenging Issues in the Literature

**DOI:** 10.7759/cureus.3732

**Published:** 2018-12-14

**Authors:** Eirini V Pantiora, Dimitrios Massaras, John Koutalas, Aikaterini Melemeni, Georgios P Fragulidis

**Affiliations:** 1 Surgery, Aretaieio Hospital, National and Kapodistrian University of Athens School of Medicine, Athens, GRC; 2 Anesthesiology, Aretaieio Hospital, National and Kapodistrian University of Athens School of Medicine, Athens, GRC

**Keywords:** pseudomyxoma peritonei, cytoreductive surgery, intraperitoneal hyperthermia, appendiceal neoplasm, classification, hipec

## Abstract

Pseudomyxoma peritonei (PMP) is a rather uncommon syndrome in oncology with a unique biological behavior and an estimated incidence of one to two cases per million per year. Clinically, it usually presents with a variety of unspecific signs and symptoms including abdominal pain and distention, ascites, or even bowel obstruction. Despite its intimidating clinical manifestation, PMP is characterized by satisfactory survival rates when treated with cytoreduction and hyperthermic intraperitoneal chemotherapy (HIPEC).

We present two interesting cases of PMP deriving from the appendix with a rather atypical presentation, which was successfully treated with cytoreduction and HIPEC. In addition, we intend to raise clinical suspicion on the diagnosis of PMP and comment on several challenging issues concerning the origin and classification of PMP.

## Introduction

Pseudomyxoma peritonei (PMP) is a rare clinical entity with an estimated incidence of one to two cases per million per year [1]. It is characterized by the dissemination of mucinous tumor deposits on peritoneal surfaces and mucinous ascites throughout the peritoneal cavity resulting in the so-called “jelly belly”. PMP predominantly originates in the appendix in men, and emerging evidence supports the appendiceal rather than ovarian origin in females. Even so, a pre-existing intraperitoneal mucinous neoplasm has been implicated as the primary cause of PMP [2].

The distribution of mucus within the peritoneal cavity occurs according to the redistribution phenomenon. Mucus and epithelial cells follow the normal course of peritoneal fluid, thus, accumulating in predetermined areas such as the omentum, paracolic gutters, and the pelvis [3,4]. Clinical presentation is related to the increased abdominal pressure caused by ascites and is often atypical, thus delaying the diagnosis. Optimal treatment is managed via complete cytoreduction and hyperthermic intraperitoneal chemotherapy (HIPEC) with satisfying results in survival [5,6].

We present the management of two male patients with PMP treated with cytoreduction and HIPEC in our department and discuss several challenging issues concerning the origin and classification of PMP.

## Case presentation

**Figure 1:**
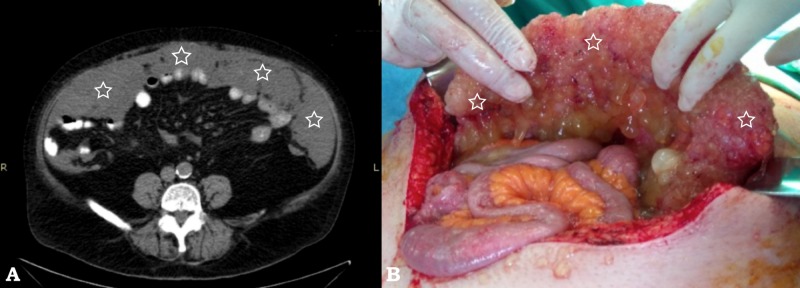
Pseudomyxoma peritonei, (patient #1) (A) Axial computed tomography (CT) slice demonstrates a large hypodense lobulated omental mass (white stars) and (B) demonstrates a corresponding perioperative view of the greater omentum with extensive tumor seeding (white stars)

The first patient was an asymptomatic male aged 59 years with a history of peripheral and autonomic neuropathy. Findings compatible with PMP were discovered in routine radiologic follow-up. Computed tomography (CT) demonstrated mucin material covering the omentum and extending in both paracolic gutters and the pelvis (Figure 1).

Serum carcinoembryonic antigen (CEA) was 210 ng/ml (normal value (nv) <5 ng/ml), while cancer antigen 19-9 (CA 19-9) was within normal range. A CT-guided biopsy was performed, which revealed mucin-producing cells of low dysplasia within mucin pools. Immunochemistry markers cytokeratin 20 (CK 20), CEA, and homeobox protein CDX-2 were positive while cytokeratin 7 was negative.

**Figure 2:**
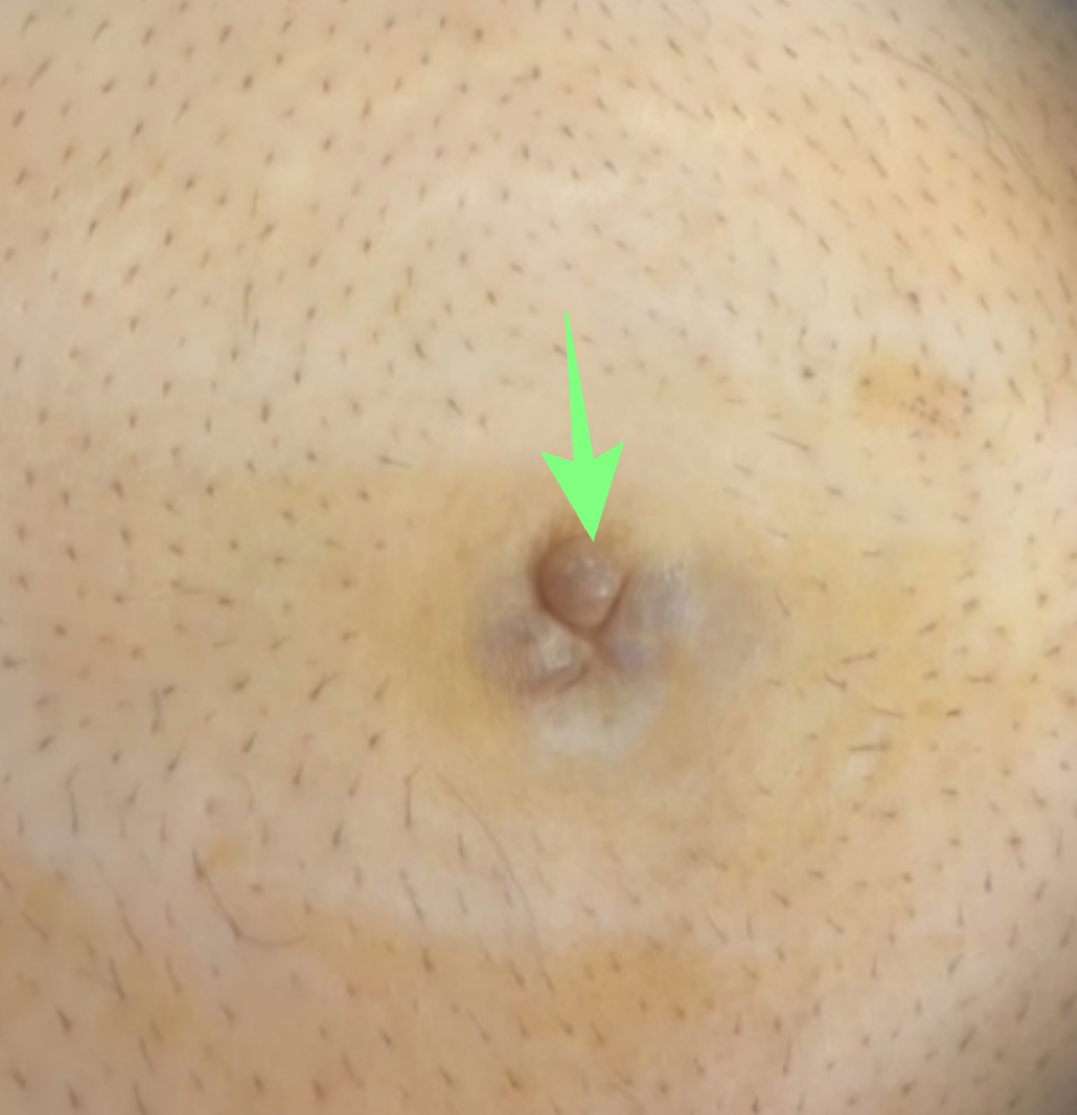
Umbilical nodule Pseudomyxoma peritonei: Umbilical nodule presented in patient #2 (arrow)

The second patient was a 62-year-old male who presented with a chief complaint of bowel movement disorders for over a year. On clinical examination, a subcutaneous, non-tender umbilical nodule was noted with no cough impulse, as can be seen in Figure 2.

**Figure 3:**
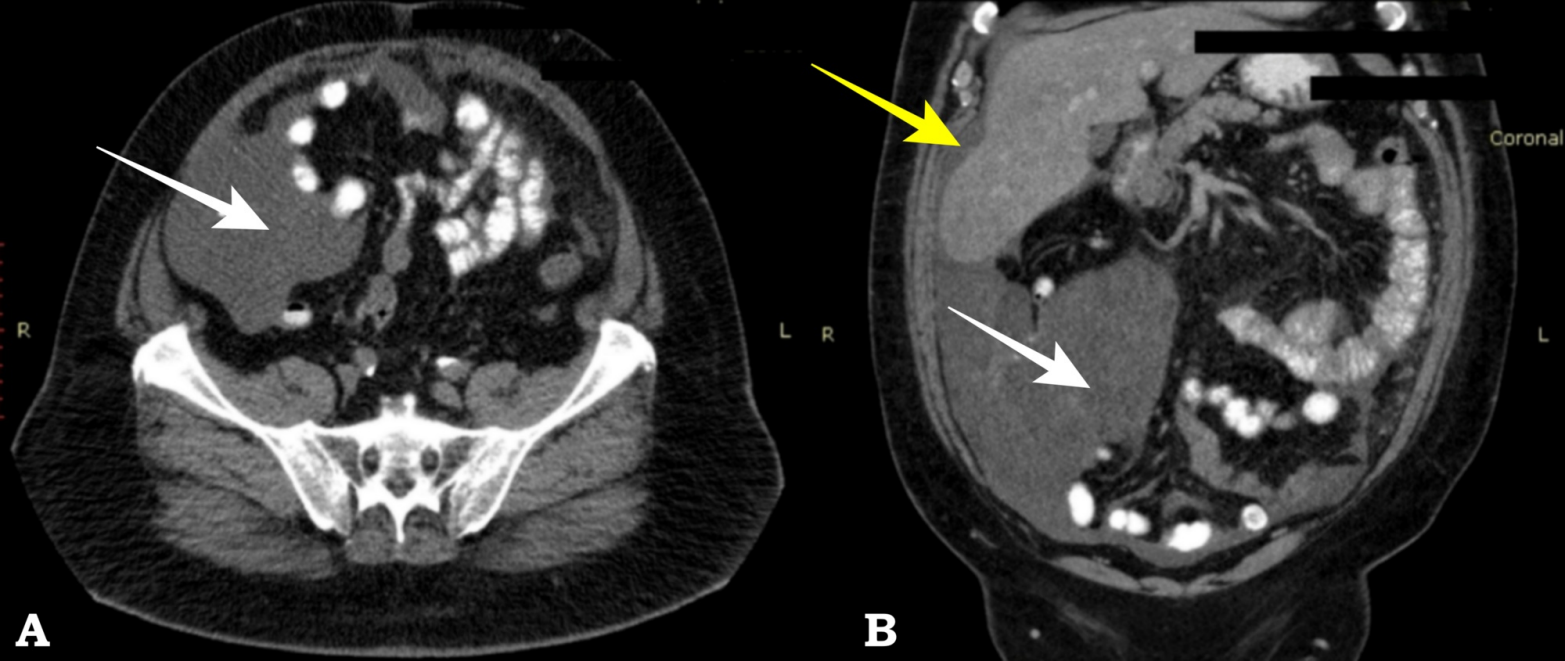
Pseudomyxoma peritonei, (patient #2) Axial computed tomography (CT) (A) and coronal CT reconstruction (B) show extensive low density tissue involving the right lower abdominal quadrant (white arrow) and perihepatic space (yellow arrow)

A CT scan demonstrated a cystic mass in the right lower quadrant with an unclear correlation to the appendix along with an ascitic collection of the right hypochondrium and paracolic gutter with septae (Figure 3).

Cytology of the ascitic fluid revealed acellular mucin without malignant component. Serum tumor markers CEA and CA 19-9 were within normal values.

**Figure 4:**
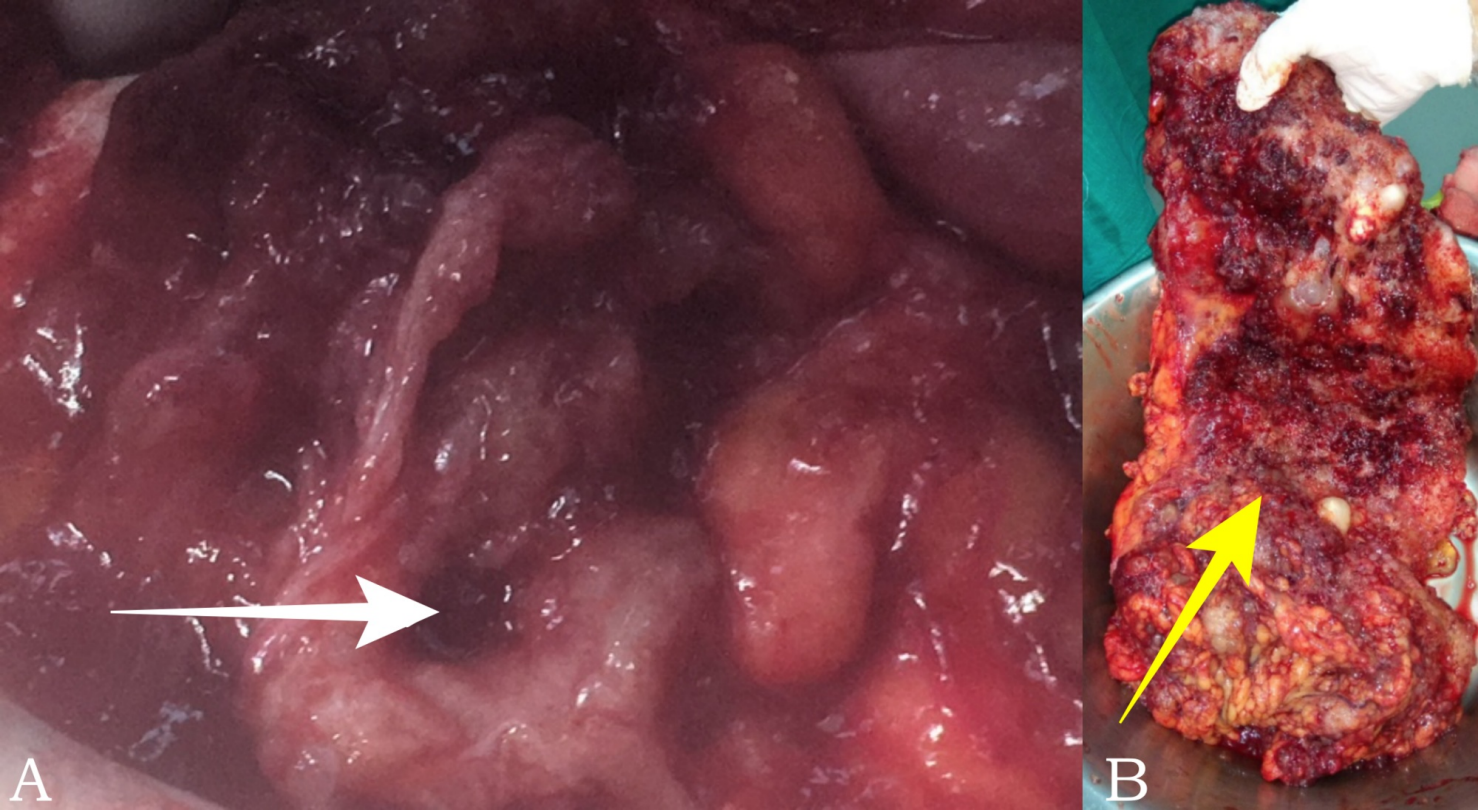
Pseudomyxoma peritonei, (patient #2) (A) Perioperative view of cecum perforation due to amputed appendix (white arrow) and (B) surgical specimen of omentectomy (yellow arrow)

Both patients underwent complete cytoreductive surgery (CRS) and HIPEC. During the second patient’s laparotomy, a ruptured cecum was discovered along with extensive mucinous ascites; therefore, he was subjected to right colectomy (Figure 4).

Next, HIPEC followed for 45 minutes using oxaliplatin in a dose of 460 mg/m2 combined with intravenous 5 fluorouracil (400 mg/m2) and leukovorin (20 mg/m2). The postoperative course was uncomplicated in both patients, and they were discharged on the tenth and seventh postoperative day, respectively. The pathology report revealed low-grade mucinous neoplasm of the appendix in the first patient and low grade with sites of high-grade dysplasia mucinous neoplasm of the appendix in the second patient. The classification of the specimens was according to the latest consensus by the Peritoneal Surface Oncology Group International (PSOGI) [7]. Both patients were free of disease in their second-year follow-up.

## Discussion

First described in 1884 by Werth, PMP remains to this day a rather challenging clinical syndrome with a unique biological behavior [8]. The true prevalence of PMP is yet unclear and is estimated to be one to two cases per million according to a study conducted in the Netherlands by Smeenk et al. [1]. Another study performed by van der Heuvel estimated its incidence in 0.6-1.9 per million in women and 0.4-1 per million in men. However, this study included only mucinous adenocarcinomas of the appendix, failing thus to cover the whole spectrum of neoplasms that PMP encompasses [9].

PMP is generally considered to derive from mucinous neoplasms of the appendix. However, various cases have been reported of syndromes resembling PMP originating from ovarian tumors, colon, pancreas, and even urachus [7]. When PMP develops from ovarian neoplasms, this is considered to be a mature teratoma within which a mucinous neoplasm has developed [10]. Besides, various studies using immunohistochemical and molecular data support the theory that ovarian neoplasms related to PMP are secondary metastases from the appendix [11]. The establishment of MUC-2, a mucin produced by goblet cells of the appendix, as a specific marker of PMP further supports the theory of the appendiceal origin of this entity [12]. Mucinous appendiceal neoplasms that lead to PMP are rare epithelial neoplasms that are included in the group of appendiceal mucoceles. Their malignant potential varies from appendiceal adenomas to adenocarcinomas.

The unique biological behavior of PMP renders its classification a rather challenging task. Several classification systems have been proposed since 1995 when Ronnet et al. proposed a system dividing PMP in three categories: disseminated peritoneal adenomucinosis (DPAM), peritoneal mucinous carcinomatosis (PMCA) and peritoneal mucinous carcinomatosis with intermediate or discordant features (PMCA-I/D) [13]. Ten years later, a study by Pai and Longacre, which distinguished four categories of appendiceal neoplasms, proposed another system: mucinous adenoma, mucinous neoplasm of uncertain malignant potential (M-UMP), mucinous neoplasm of low malignant potential (M-LMP), and mucinous carcinoma [14]. In 2006, Bradley et al. modified the initial criteria of Ronnett by unifying DPAM and PMCA-I and by including the cases expressing signet cell in the PMCA group [15]. In 2010, the World Health Organization (WHO) launched a new, simplified two-tier system of low grade and high-grade pseudomyxoma. The latest classification available was published in 2016 by PSOGI and was conducted using the modified Delphi process. According to this, only lesions with infiltrative invasion are called mucinous adenocarcinoma. Lesions without infiltrative invasion but with high-grade cytologic atypia are characterized as high-grade appendiceal mucinous neoplasms. The term low-grade appendiceal mucinous neoplasms describe mucinous neoplasms with low-grade atypia. Neoplasms with signet ring are classified as poorly differentiated mucinous adenocarcinoma with signet ring cells when those comprise less than 50% of the tumor and as mucinous signet ring cell carcinoma when more than 50% of the cells show signet ring morphology. PMP is classified as low-grade mucinous carcinoma peritonei, high-grade carcinoma peritonei, and high-grade carcinoma with signet ring cells. Acellular mucin is a different category [7]. Despite the fact that this new classification reflects a well-organized effort to clarify and unify the terminology concerning PMP classification, several confusing issues such as the different classification used for the primary neoplasia and the consequent peritoneal disease impede its universal acceptance. A validation study by Baratti et al., failed to confirm the superiority of the PSOGI classification as a prognostic tool over the WHO classification and suggests the need for further studies [6].

The American Joint Committee on Cancer (AJCC) issued in 2017 the eighth edition of grading and staging of appendiceal tumors. This system is focused on clarifying the grading of low-grade appendiceal mucinous neoplasms (LAMN) according to tumor extension and introduces the term in situ for LAMN confined in the muscularis propria. In accordance to the PSOGI classification, the AJCC also proposes a three-tier system of classification of disseminated appendiceal neoplasms, as this seems to have a prognostic significance [16].

Diagnosis of PMP is quite challenging due to the varied clinical presentation of the disease. It often presents as acute appendicitis and is an incidental finding during laparotomy or laparoscopy for suspected appendicitis, peritonitis, or gynecological cancer. In more progressed cases, it may manifest as increased abdominal girth due to the mucinous ascites or a new onset hernia as a result of increased abdominal pressure. In women the most common manifestation is that of an ovarian mass [17]. In our cases the first patient was asymptomatic and the second described a vague complaint of irregular bowel movements, verifying the multifaceted manifestation of PMP.

CT scan is the gold standard in the diagnosis of PMP. Low attenuation mucinous ascites with possible septae or calcifications is seen particularly in the paracolic gutters and the pelvis, along with omental implants. CT imaging of mucus “scalloping” of the liver capsule is typical and it was present in both of our patients, as can be seen on Figures 1 and 3 [18]. Serum tumor markers have a prognostic role and are useful in postoperative follow-up, but are not particularly useful or specific for the diagnosis of PMP.

Treatment of PMP is best managed with cytoreductive surgery combined with HIPEC with reported five-year survival rates reaching up to 86% [19]. Systemic chemotherapy without the use of intraperitoneal chemotherapy is not advisable due to the inability of chemotherapeutic agents to achieve adequate levels in mucin-surrounded tumors [20]. Repetitive debulking used to be the standard treatment in the previous years, but through the introduction of HIPEC in the field of surgical oncology, complete CRS along with HIPEC has been established as the treatment of choice [7, 17].

## Conclusions

In conclusion, PMP is a clinical syndrome describing a highly complex and rare entity, whose biological behavior and pathophysiological pathways are yet to be defined. Molecular research and well-organized clinical studies have managed important steps towards the clarification of this disease, yet more extensive studies need to be performed. Untangling issues concerning the origin and molecular pathways of PMP may provide new, targeted therapy options. A simplified, universal classification system should also be established in order to facilitate communication among physicians. The satisfying outcomes provided by CRS and HIPEC dictate further development in the fields of both diagnosis as well as treatment.
